# Structure of Haze Forming Proteins in White Wines: *Vitis vinifera* Thaumatin-Like Proteins

**DOI:** 10.1371/journal.pone.0113757

**Published:** 2014-12-02

**Authors:** Matteo Marangon, Steven C. Van Sluyter, Elizabeth J. Waters, Robert I. Menz

**Affiliations:** 1 The Australian Wine Research Institute, Adelaide, South Australia, Australia; 2 School of Biological Sciences, Flinders University, Adelaide, South Australia, Australia; 3 School of Botany, University of Melbourne, Victoria, Australia; Università di Napoli Federico II, Italy

## Abstract

Grape thaumatin-like proteins (TLPs) play roles in plant-pathogen interactions and can cause protein haze in white wine unless removed prior to bottling. Different isoforms of TLPs have different hazing potential and aggregation behavior. Here we present the elucidation of the molecular structures of three grape TLPs that display different hazing potential. The three TLPs have very similar structures despite belonging to two different classes (F2/4JRU is a thaumatin-like protein while I/4L5H and H2/4MBT are VVTL1), and having different unfolding temperatures (56 vs. 62°C), with protein F2/4JRU being heat unstable and forming haze, while I/4L5H does not. These differences in properties are attributable to the conformation of a single loop and the amino acid composition of its flanking regions.

## Introduction

Securing wine stability is essential in winemaking. Among the possible instabilities that can occur, protein haze formation is the most important instability of non-microbial origin, particularly for white wine production [1], [2]. *Vitis vinifera* grape juices and wines generally contain between 10 and 500 mg/L of protein [3]. Despite these low concentrations, proteins have a great technological relevance in winemaking as some of them, the grape pathogenesis-related (PR) proteins, can aggregate to form a visible haze [4], [5]. The grape PR proteins [2] are constitutively expressed in healthy plants, and are further expressed in response to biotic or abiotic stresses [6].

Among the grape PR-proteins, the thaumatin-like proteins (TLPs) and chitinases can undergo changes in structural integrity, particularly under certain conditions such as when wines are exposed to elevated temperatures during storage or transportation. These changes lead to protein unfolding, resulting in the exposure of amino acid side chains that are normally hidden in the core of the protein. The newly exposed side chains are then free to associate with neighbouring proteins or with other wine components to form aggregates that result in visible haze or precipitates in the bottles [7].

Hazy wines are not saleable because consumers perceive them as faulty, and therefore proteins need to be removed before bottling, a step achieved by addition of bentonite, a clay that is negatively charged at wine pH thus binding to the positively charged wine proteins and settling to the bottom of the tanks [2]. The use of bentonite is costly and has several quality drawbacks [8], so that alternatives are sought, but in order to find a valid substitute for bentonite, a better understanding of the role played by the grape thaumatin-like proteins and chitinases in the mechanism of protein haze formation is required.

Research on wine proteins traditionally has treated chitinases and TLPs in the same way because both classes are normally associated with haze formation. However, since the development of an efficient purification method for the wine proteins [9] there has been an increasing interest in the characterisation of these two classes of proteins. A major finding from comparative studies is that chitinases are more heat unstable and more prone to aggregate than TLPs [10], [11]. In addition, heat unfolded chitinases cannot refold back to a native state, while TLPs can [10]. However, it has recently been reported that some grape TLPs do not follow this behaviour [12]–[14], and these observations combined with reports on the presence of both TLPs and chitinases in wine hazes [2], [15], [16], indicate that some TLPs can also contribute to haze formation.

TLPs have been well characterised in several plants, and their structure has been determined for eight plant TLPs, namely thaumatin [17], zeamatin [18], tobacco PR-5d [19], osmotin [20], the cherry allergen Pru Av2 [21], banana TLP [22], tomato NP24-I [23], apple allergen Mal d 2 [24], and kiwi-fruit allergen act d 2 [25]. Plant TLPs generally have three domains and a cleft located between domains I and II. Domain I is the central core of the protein and is formed by several β-strands; several α-helixes are located in domain II, while domain III is comprised of β-strands and small loops. Each domain is stabilized by at least one disulfide bridge formed by up to 16 cysteine residues with a conserved spatial distribution throughout the protein [20]. The cleft between domains I and II may have an acidic, neutral, or basic nature for binding different ligands/receptors. All PR5 proteins known to have antifungal activity in plants are characterised by an acidic cleft, due to the presence of five highly conserved amino acids (arginine, glutamic acid, and three aspartic acid residues). This feature is found in grape TLPs and likely confers antifungal activity against pathogens such as *Phomopsis viticola* and *Botrytis cinerea* [26].

To date, the structures of grape TLPs have not been elucidated. Since the accurate determination of protein structures provides insights into their function [27], the availability of crystallographic data for the haze forming proteins could lead to the elucidation of the mechanism of haze formation phenomena driven by grape TLPs. It could also assist with the development of proteolytic enzymes able to cleave these proteins so to prevent their aggregation during winemaking.

In this paper the crystal structure and some physico-chemical parameters relevant for the haze formation mechanism of three isoforms of TLPs isolated from grape juice were determined. The solved structures were compared in order to understand the structural basis for the different hazing potential of these grape thaumatin-like proteins.

## Materials and Methods

### Protein purification

The proteins used were purified from Sauvignon blanc grape juice sourced in 2006 from the Adelaide Hills region (South Australia) as previously described [9] (Table S1).

### Crystallization

Prior to crystallization the proteins were concentrated (Table S2). Initial crystallization conditions for the three proteins were screened by the sitting-drop vapor-diffusion method using commercially available sparse-matrix screening kits (Nextal suite, Qiagen). The 96 well microintelliplates were loaded by means of a robot (Phoenix). Pictures of each well were taken daily in order to monitor the crystals growth. The wells indicating crystal formation were selected for scaling-up into 24 well intelliplates. The drops contained 0.5 µL of stock protein mixed with 0.5 µL of the precipitant solution (500 µL of reservoir). After this scale-up, further finer screening around the conditions identified was carried out to identify the optimum conditions (Table S2).

### Data collection and processing

Diffraction data were collected from single cryo-cooled (100°K) crystals that were robotically mounted on beamlines MX1 (for protein F2/4JRU and I/4L5H) or MX2 (for protein H2/4MBT), controlled using Blu-Ice software [28] at the Australian Synchrotron, Victoria, Australia (see Table 1). Diffraction images were collected on Area Detector Systems Corp. CCD detectors, and diffraction data were analyzed using MOSFLM interfaced by iMOSFLM version 1.0.7 (using MOSFLM 7.0.9) [29].

**Table 1:** Data collection and processing.

	F2/4JRU	I/4L5H	H2/4MBT
Diffraction source	Australian synchrotron beamline MX1	Australian synchrotron beamline MX1	Australian synchrotron beamline MX2
Wavelength (Å)	0.956639	0.956639	0.97946
Temperature (K)	100	100	100
Detector	ADSC quantum 210R CCD	ADSC quantum 210R CCD	ADSC quantum 210R CCD
Crystal-detector distance (mm)	125.00806	149.96144	200.06950
Rotation range per image (°)	0.25	1.00	0.50
Total rotation range (°)	219–309	253–353	0–120
Exposure time per image (s)	0.75	0.75	0.50
Space group	P2_1_2_1_2_1_	C2	C2
Unit-cell parameters *a, b, c* (Å)	34.80, 70.17, 74.90	122.21, 52.73, 94.39	122.56, 52.60, 91.61
α, β, γ (°)	90, 90, 90	90, 132.23, 90	90, 130.02, 90
Average mosaicity (°)	0.45	1.22	0.50
Resolution range (Å)	14.907–1.200 (1.26–1.20)	12.630–1.590 (1.67–1.59)	23.460–1.600 (1.69–1.60)
Total No. of reflections	156606	112205	131964
No. of unique reflections	51588	45266	53130
Completeness (%)	88.4 (49.6)	75.7 (78.2)	90.2 (94.3)
*I/σ (I)* (overall) (%)	16.1 (7.8)	8.5 (2.1)	6.6 (1.8)

Values for the highest resolution shell are shown in parentheses.

### Structure solution and refinement

The programs MOSFLM [30] and SCALA [31] were used for data reduction (Table 2). The structure of protein F2/4JRU was solved by molecular replacement using the program *MOLREP* [32] and refined with REFMAC 5.6.0117 [33] implemented in the CCP4 program suite 6.2.0 (Collaborative Computational Project) using a search model prepared from the thaumatin-like protein from banana (PDB ID: 1Z3Q) using CHAINSAW [34]. Proteins I (PDB ID: 4L5H) and H2 (PDB ID: 4MBT) were similarly solved except that the F2 structure (PDB ID: 4JRU) and the I structure were used as the search model, respectively. *MolProbity* [35] was used for Ramachandran analysis. Models were validated with PROCHECK [36]. Molecular images were created by UCSF Chimera version 1.7 [37]. The structures have been deposited in the Protein Data Bank under the IDs 4JRU (F2), 4L5H (I), and 4MBT (H2).

**Table 2:** Structure refinement.

	F2/4JRU	I/4L5H	H2/4MBT
Resolution range (Å)	14.62–1.20 (1.23–1.20)	12.63–1.80 (1.85–1.80)	8.92–1.65 (1.69–1.65)
Completeness (%)	88.3	74.5	88.9
σ cutoff	2.0	2.0	2.5
No. of reflections, working set	48915 (1632)	29314 (2133)	45549 (3307)
No. of reflections, test set	2608 (81)	1596 (108)	2457 (203)
Final R_cryst_	0.166 (0.194)	0.198 (0.220)	0.224 (0.315)
Final R_free_	0.184 (0.237)	0.233 (0.275)	0.257 (0.394)
No. of non-H atoms: Protein	1483	2972	2972
No. of non-H atoms: Ligand	18	6	6
No. of non-H atoms: Solvent	201	181	156
No. of non-H atoms: Total	1763	3226	3213
R.m.s. deviations: Bonds (Å)	0.027	0.023	0.027
R.m.s. deviations: Angles (°)	2.345	2.221	2.284
Average *B* factor (Å^2^): Protein	9.5	12.1	17.3
Average *B* factor (Å^2^): Ligand	21.8	20.8	17.1
Average *B* factor (Å^2^): Water	19.1	23.2	22.5
Ramachandran plot: Favored regions (%)	97.0	97.7	97.2
Ramachandran plot: additionally allowed (%)	2.5	2.0	2.5

Values for the outer shell are given in parentheses.

### Heat Test

Purified proteins stored as ammonium sulphate suspensions were centrifuged (13000 *g*, 15 min, 4°C), and the pellets dissolved in deionized water. Salt removal and protein concentration were achieved via centrifugation with Nanosep 3 kDa ultrafiltration devices (Pall Corp., USA). Concentrated proteins were dissolved in model wine (12% ethanol, 2 g/L, malic acid, 1 g/L K_2_HSO_4_, pH 3.20) at 50 mg/L concentration. Samples were heated at 80°C for 2 h and cooled at 4°C for 2 h. After equilibration to ambient temperature, the haze was measured by calculating the difference between the heated and unheated samples in the absorbance values at 540 nm [38] by means of a spectrophotometer (Beckman DU 530 Life Science UV-vis spectrophotometer, Beckman Coulter, Fullerton, CA) [39].

### Protein HPLC and Sodium Dodecyl Sulfate-Polyacrylamide Gel Electrophoresis (SDS-PAGE)

Proteins were analyzed by RP-HPLC and by SDS-PAGE as described previously [11].

## Results

**Figure 1:**
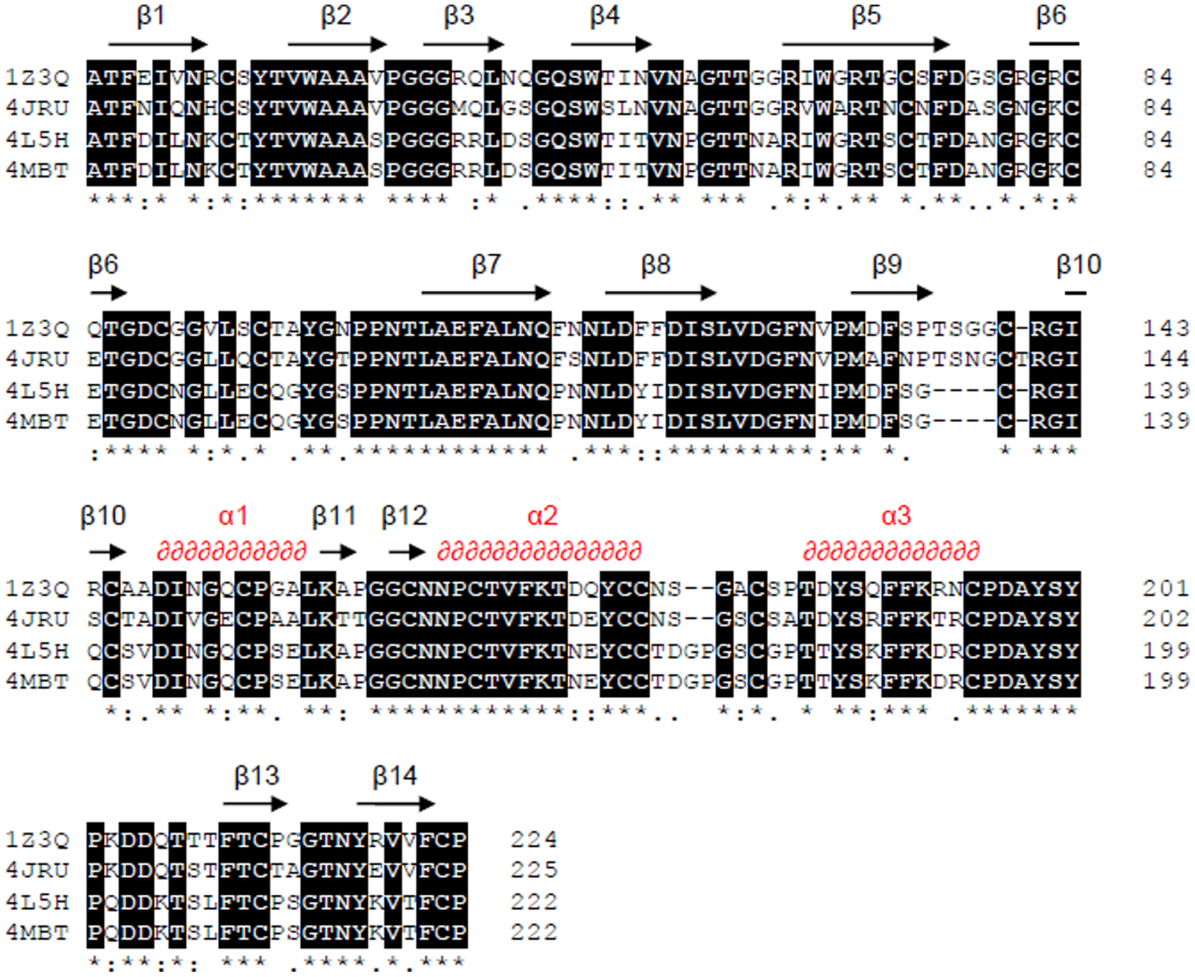
Alignment of amino acid sequences of Banana thaumatin-like protein (1Z3Q from *Musa acuminata*) and of the three *Vitis vinifera* thaumatin-like proteins F2/4JRU, I/4L5H, and H2/4MBT. The multiple alignments were performed with the alignment function of the STRAP software [55]. The overall secondary structure for the fold predicted by STRAP software is shown.

The three thaumatin-like proteins isolated from *Vitis vinifera* shared significant sequence similarity to the banana thaumatin-like protein (1Z3Q), the structure of which has been solved [22]. The pairwise protein alignment showed that the banana-TLP had 79.6% identity with F2/4JRU, and 72.3% identity with proteins I/4L5H and H2/4MBT, while protein F2/4JRU had 69% identity with proteins I/4L5H and H2/4MBT (Figure 1, Table S3). It should be noted that proteins H2/4MBT and I/4L5H, despite sharing the same sequence have been purified based on their different physical properties [9].

Due to the high sequence similarity, the banana structure was used to determine the structure of protein I/4L5H by molecular replacement, which was the first *Vitis vinifera* protein for which we obtained diffraction data. The initial solution for this structure was deposited in the Protein Data Bank as 4H8T. This initial structure included data to 2.0∶Å and an overall R value of 0.224. Subsequently, the structure of 4H8T has been used as a search model to solve the structure of protein F2/4JRU, for which we had data at atomic resolution (1.2∶Å, see Table 2), and which is now listed on the Protein Data Bank under the ID 4JRU. Protein F2/4JRU was then used as a search model to re-process the dataset from which we obtained our first structure (4H8T), resulting in a significant improvement of its solution (overall R = 0.200, resolution 1.8∶Å) that was re-deposited to the Protein Data Bank under a new ID (4L5H, Table 2). The third crystal from which we had x-ray diffraction data was protein H2, a protein that shares the same sequence as protein I. Protein H2 was solved using I/4L5H as a search model. The crystals of H2 were much smaller than those of I, hence diffraction data for these crystals were collected on the micro-crystallography beamline (MX2, Table 1). Data were obtained up to 1.5∶Å, however there was a noticeable decrease in the quality of the last 60 images collected (out of 240), likely due to radiation damage, obliging us to cut the spot recognition to 2.5 σ (see Table 2), thus limiting the resolution to 1.65∶Å. The structure is now deposited to the PDB under the ID 4MBT.

The protein folds of the 3 grape TLP structures were analyzed by CATH tool (http://www.cathdb.info/search/by_structure) [40], and compared to the banana TLP. As expected, due to their high sequence similarity (Figure 1, Table S3), all four proteins belong to the Thaumatin superfamily fold (CATH 2.60.110.10), and within this superfamily they belonged to 2 different functional families: proteins 1Z3Q and F2/4JRU have a Thaumatin-like protein-like domain, while I/4L5H and H2/4MBT have a Pathogenesis-related protein-like domain. These differences are reflected in the protein alignment as shown by the gaps observed between β-sheets 9 and 10, and α-helixes 2 and 3 (Figure 1).

### Structure description of protein F2/4JRU

**Figure 2:**
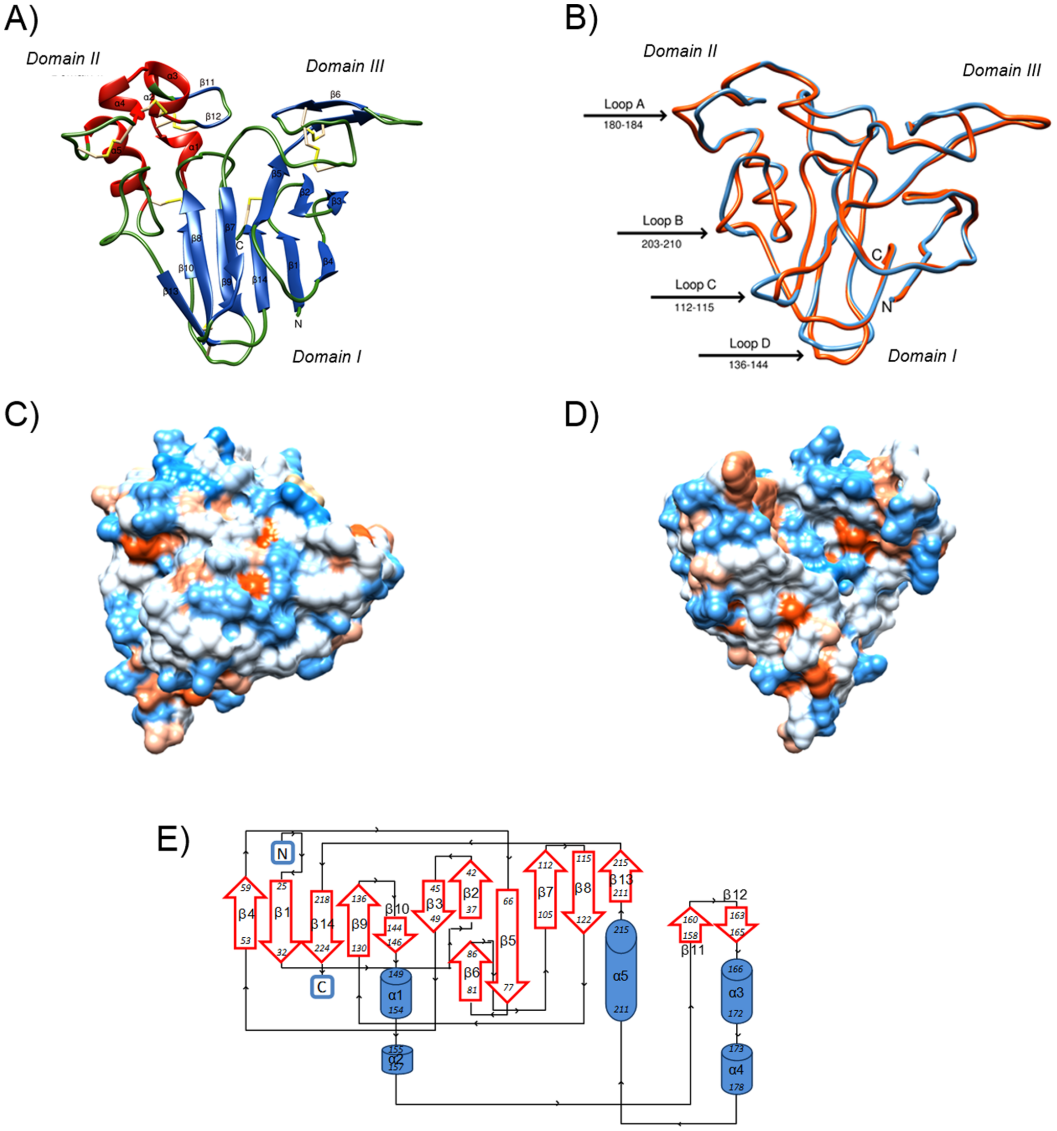
Three-dimensional structure of protein F2/4JRU. A) Ribbon diagram of the overall three-dimensional structure of protein F2/4JRU. The protein consists of three structural domains: a central core domain I built from a β-sandwich of two sheets of six (front) and five (back) β-strands, flanked by two shorted domains II (where the 5 α-helixes and β-strand 11 and 12 are located) and III (where two β-strands - 5 and 6 - and a turn form two looping regions). The eight disulphide bridges are shown in yellow. B) Superposition of the backbone representations of the grape TLP F2/4JRU (in orange) and banana TLP 1Z3Q (in blue). TM-align data showed that the two proteins have RMSD of 0.78 Å, and a TM-score of 0.98 [41]. Significant differences in loops' structures are indicated by arrows, and numbers denote residues in F2/4JRU. C) and D) Mapping of the surface hydrophobicity of the grape TLP F2/4JRU, front view (same as Figure 2A) and back view (rotated approximately 180°) respectively. Hydrophobicity continuum from orange to blue representing hydrophobic to hydrophilic. E) Topology of protein F2/4JRU showing the residues included in each β-strand or α-helix.

The crystal structure of F2/4JRU was determined at 1.20∶Å resolution (Table 2 and Figure 2).

Protein F2/4JRU, similar to other thaumatin-like proteins [22], [23], presents three domains (Figure 2A). Domain I is the largest and forms the central core of the molecule. This domain comprises 11 β-strands forming a β-sandwich of two β-sheets. In particular, six anti-parallel β-strands form the front sheet (β2, β3, β5, β7, β8, and β13), and the other five form the back sheet (β1, β4, β9, β10, and β14), which together constitute the β-sandwich. The two β-sheets are stabilized by four disulfide bonds (residues 33–224, 89–95, 140–213, and 146–196). Domain II consists of a long α-helix (α5) and four α-helical segments (α1 to α4), and is stabilized by four disulfide bonds (residues 146–196, 154–164, 168–177, 178–183). The short domain III is made up of a stretch of 32 amino acid residues (residues 72–104) comprising a long loop and 2 β-strands (β5, long strand in common with domain I, and β6). This domain is stabilized by two disulfide bonds (residues 74–84, 89–95). Importantly, disulfide bond 89–95 connects domain I to III, while disulfide bond 146–196 connects domain I to II. All the eight disulfide bonds, marked in yellow in Figure 2A, are highly conserved in thaumatin [17] and the TLPs [18], [19], [22]. Overall, protein F2/4JRU is composed of 14 β-strands and 5 α-helixes as displayed in the protein topology diagram (Figure 2E).

In agreement with the results of the sequence alignment (Figure 1), the superposition of 1Z3Q and 4JRU shows that the structures have a high degree of similarity, with only four differing loop regions (Figure 2B). The comparison of the two structures performed with the algorithm for protein structure alignment and comparison named TM-align tool [41] indicated that the two proteins have a root mean square deviation (RMSD) of 0.78 Å, with a TM-score of 0.98, indicating that the proteins belong to the same fold (thaumatin), confirming results previously obtained with CATH. The 4 differing regions are indicated by the arrows as shown in Figure 2B. Differences were observed in loops between helixes α4 and α5 (loop A), between α5 and β13 (loop B) between β7 and β8 (loop C), and between β9 and β10 (loop D). These differences relate to alternate conformations of the loops between the two structures, rather than differences in the lengths of the loops (Figure 1 and Figure 2).

The surface of the protein (F2/4JRU) is predominantly hydrophilic supporting the physical properties observed for the purified protein (Table 3 and Figure 2C and 2D).

**Table 3:** Summary of the physical properties of F2/4JRU, I/4L5H and H2/4MBT.

	F2/4JRU	I/4L5H	H2/4MBT
SCX elution conditions a	22 mM sodium citrate pH 3.0,	30 mM MES pH 6.0,	14 mM sodium citrate pH 3.0,
	8 mM MES pH 6.0,	1 M NaCl	16 mM MES pH 6.0, 0.3 M NaCl
	0.27 M NaCl		
HIC elution conditions a	pH 5.0,	pH 5.0,	pH 5.0,
	0.50 M Amm Sulphate	0.70 M Amm Sulphate	0.71 M Amm Sulphate
Molecular Weight	21252	21287	21287
Measured mass a	21241	21275	21275
RP-HPLC RT a	12.6 min	15.1 min	15.1 min
Isoelectric point (p*I*) b	4.54	4.76	4.76
Unfolding temperature T*m* (°C)^c^	56	62	62
Thermal unfolding behavior c	Partial reversibility	Partial reversibility	Partial reversibility

a data from Van Sluyter et al., 2009.

b shown values are calculated, not experimental.

c data from Falconer et al., 2010.

**Figure 3:**
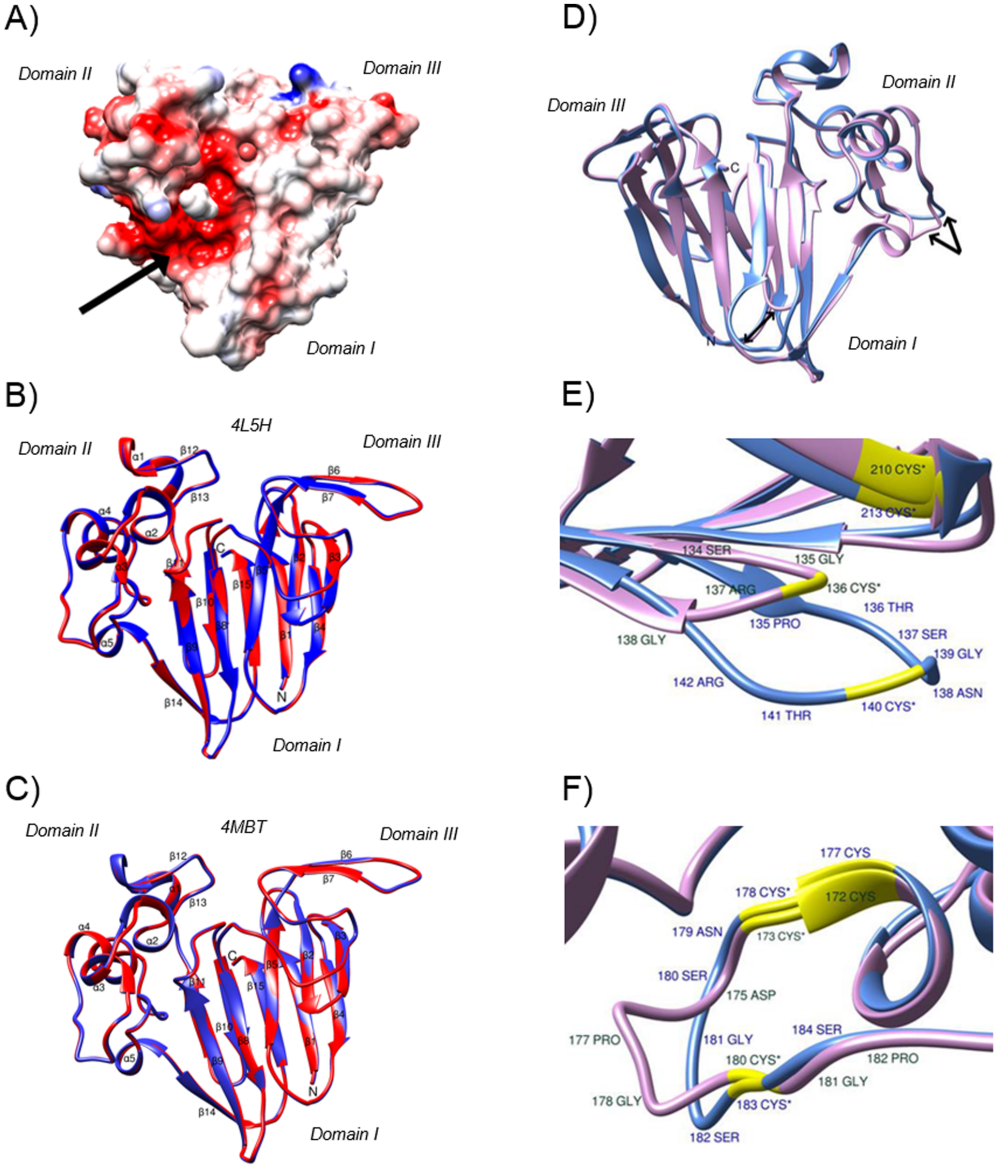
Comparison of the three-dimensional structures of grape TLP. A) Mapping of the electrostatic potentials on the molecular surface of the grape TLP F2/4JRU. The negative potentials and positive potentials are colored red and blue, respectively. Neutral surfaces are white. Arrow indicates the acidic cleft region located in between domains I and II. B) Superposition of the backbone representations of chain A (in red) and B (blue) of I/4L5H. TM-align data showed that the two chains of I/4L5H have RMSD of 0.14 Å, and a TM-score of 0.99925. C) Superposition of the backbone representations of chain A (in red) and B (blue) of H2/4MBT. TM-align data showed that the two chains of H2/4MBT have RMSD of 0.13 Å, and a TM-score of 0.99939. D) Superposition of the secondary structure of I/4L5H chain A (in purple) and F2/4JRU (in blue). Arrows indicate the two loop regions showing the largest differences between the two structures. RMSD between 193 atom pairs is 0.505 Å (calculated with UCSF Chimera MatchMaker function). E) Detail of differences in loop regions in Domain I. F) Detail of differences in loop region in Domain II. Cysteine are shown in yellow, and disulfide bonds are formed between residues 140–213 and 183–178 in F2/4JRU, and residues 136–210 and 180–173 in I/4L5H, as indicated by the asterisks.

In the structure of F2/4JRU, a prominent cleft is observed between domains I and II, a characteristic feature common to thaumatin and thaumatin-like proteins. Electrostatic modelling of F2/4JRU reveals that this cleft is very acidic (Figure 3A), as also observed in the three-dimensional structures of other antifungal TLPs [18]–[20], [22]. This acidity is due to the presence of four conserved acidic residues, Glu107, Asp120, Asp125 and Asp206 in F2/4JRU, the side-chains of which intrude inside the cleft from domain II. Two of these, Glu107 and Asp120, however, are also present in thaumatin, which has a predominantly basic cleft.

### Structure description of VVTL1 protein I/4L5H and H2/4MBT

The crystal structure of two other purified *Vitis vinifera* thaumatin-like proteins (VVTL1), proteins I/4L5H and H2/4MBT, was determined at 1.80 and 1.65∶Å resolution, respectively (Table 2). Despite sharing the same sequence, the two VVTL1 isoforms, I and H2 exhibited differences in their chromatographic behavior, a feature that allowed their separation and that also indicated that they possess some differences in physical properties. In detail, protein I/4L5H eluted during SCX at pH 6.0, 1 M NaCl, while protein H2/4MBT eluted at pH 4.35, 0.3 M NaCl. During the second purification step they did not show differences in hydrophobicity, as by HIC they both eluted at pH 5.0, 0.7 M ammonium sulphate (Table 3). The physical differences between these two isoforms of the protein were also observed during crystallography. Although similar, distinctly different conditions were required to obtain crystals of the two proteins (Table S2) [9].

Unlike F2/4JRU, which belongs to space group P2_1_2_1_2_1_, the two VVTL1 crystallized in space group C2 (Table 1). This difference in space group resulted in the asymmetric unit containing a dimer of the protein. However, it is likely that the dimer is a crystallization artifact, an assumption supported by the fact that size exclusion chromatography analysis conducted to assess the possible presence of dimerisation of these proteins in solution did not give any indication of this (data not shown). This theory is also supported by the superposition analysis of chain A and B of the two VVTL1 (Figure 3), which demonstrated that both chains adopted an identical conformation with an RMSD values of 0.14 Å for I/4L5H, and 0.13 Å for H2/4MBT.

The two VVTL1 proteins, similar to protein F2/4JRU (see Figure 2A), have three domains (Figure 3B and 3C). Domain I is the largest and is made of 11 β-strands (β2, β3, β5, β8, β9, and β14 on the front; β1, β4, β10, β11, and β14 on the back), forming a β-sandwich of two β-sheets. The two β-sheets are stabilized by four disulfide bonds (residues 33–221, 89–95, 136–210, and 141–193). Domain II consists of a long α-helix (α4) and four α-helical segments (α1, α2, α3, and α5), and is stabilized by four disulfide bonds (residues 141–193, 149–159, 163–172, 173–180). The short domain III comprised 33 amino acid residues (residues 72–105), and includes a long loop and 2 β-strands (β6 and β7). This domain is stabilized by two disulfide bonds (residues 74–84, 89–95). Two disulfide bonds are shared by different domains: disulfide bond 89–95 connects domain I to III, while bond 141–193 connects domain I to II. Overall, proteins I/4L5H and H2/4MBT are composed of 15 β-strands and 5 α-helixes.

A further comparison of chains A of I/4L5H and H2/4MBT showed that they are almost identical as well since the RMSD between the two is 0.14 Å, with a TM-score of 0.99931. The solved crystal structures do not provide an explanation for their different chromatographic behavior, and this remains a topic for further study. Given the similarity of chains A of I/4L5H and H2/4MBT, only chain A of protein I/4L5H will be discussed in the following sections (Figure 3B, protein in red).

In order to visualize structural differences between grape TLPs, the 3D structure of F2/4JRU was superposed to that of chain A of I/4L5H (Figure 3D to F). From the superposition of the two proteins it is clear that there are only two regions presenting major differences (Figure 3D). These differences are caused by the presence of extra amino acids in one protein or in the other. Figure 3E shows the longer loop in Domain I between β9 and β10 of F2/4JRU, which is due to the presence of 5 extra amino acids (136 TSNGCT 141) that are not present in I/4L5H, as highlighted by the sequence alignment (see Figure 1). This results in a longer and therefore more exposed loop for F2/4JRU than I/4L5H, a characteristic that could confer different physical properties to the protein. The second region exhibiting differences is located in Domain II. In this case, I/4L5H has a longer loop (see Figure 3F), due to the presence of 2 extra amino acids between α3 and α4 in positions 176–177 (175 DGPGCS 180) as shown in Figure 1.

### Relation of protein structural features and haze forming properties

Despite the similarities in terms of sequence, fold and structure that the three grape TLPs have exhibited (Table S3, Figures 1 and 3), further investigation on their physico-chemical characteristics highlighted the presence of differences, particularly in terms of purification conditions and heat stability (Table 3).

The three proteins were purified based on the differences in their chromatographic characteristics. In particular, F2/4JRU eluted first during the SCX step probably because it is the protein with the lowest p*I* and so the least charged under separation conditions. During the HIC step F2/4JRU was eluted at 0.5 M ammonium sulphate versus 0.7 M for both I/4L5H and H2/4MBT, therefore the most hydrophobic protein of the three (Table 3).

Other observed differences were related to unfolding behaviour upon heating. Falconer et al. [10] found that grape TLPs are generally more stable than other haze-forming wine proteins such as chitinases, but it was also found that there is variation among TLP isoforms. In particular it was found that F2/4JRU has a significantly lower unfolding temperature (56°C) than I/4L5H and H2/4MBT (62°C) (Table 3), even though the three proteins demonstrated partial reversibility upon cooling.

**Figure 4:**
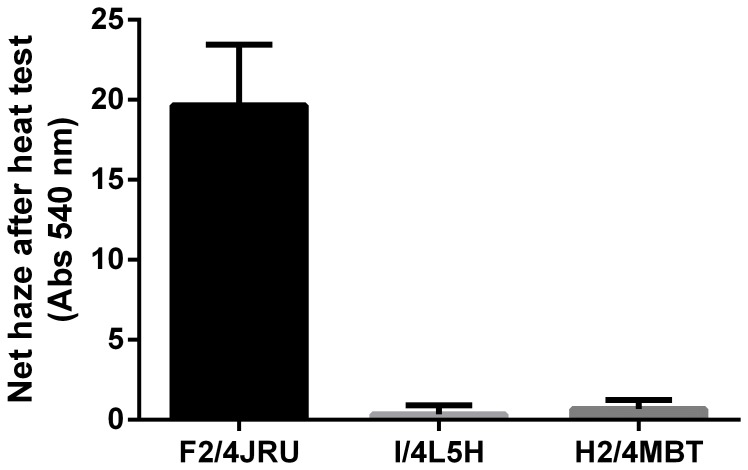
Haze developed upon heat test of proteins F2/4JRU, I/4L5H and H2/4MBT. Haze was measured after treatment at 80°C for 2 h, followed by 4°C for 2 h. Assays were carried out at 50 mg/L in model wine.

In order to study aggregation upon heat unfolding, proteins were prepared in a model wine and exposed to a heating/cooling cycle to favour protein unfolding and possible aggregation (Figure 4), mimicking the mechanism of haze formation during winemaking.

Data from the heat test indicated that aggregation was taking place only for protein F2/4JRU, while I/4L5H and H2/4MBT, despite unfolding during the test, did not form large aggregates upon cooling, thus suggesting that their degree of reversibility is higher than F2/4JRU. These data are in agreement with previous research conducted on these proteins [10], [11], [42].

**Figure 5:**
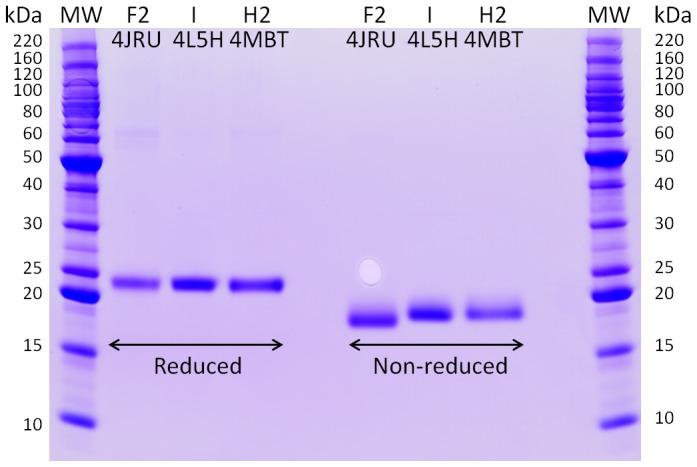
SDS-PAGE of purified proteins under reducing and non-reducing conditions. Proteins (∼2 µg per lane) were reduced with 5% BME or loaded on a 12% gel in non-reducing conditions and subjected to SDS-PAGE.

The electrophoretic mobility of the three TLPs was also investigated in both reducing and non-reducing conditions (Figure 5).

Results indicate that the mobility of the three proteins varies depending on the presence/absence of the reducing agent. When the reducing agent is present all the disulfide bridges are reduced and no differences in the migration pattern was observed, while in absence of the reducing agent F2/4JRU was more compact and therefore migrated to a lower level than the two VVTL1. This change in mobility indicates that, despite being small, the observed differences in the loops between F2/4JRU and I/4L5H (Figure 3) must be significant enough to result in a change of the mobility and compactness of proteins during SDS-PAGE.

**Figure 6:**
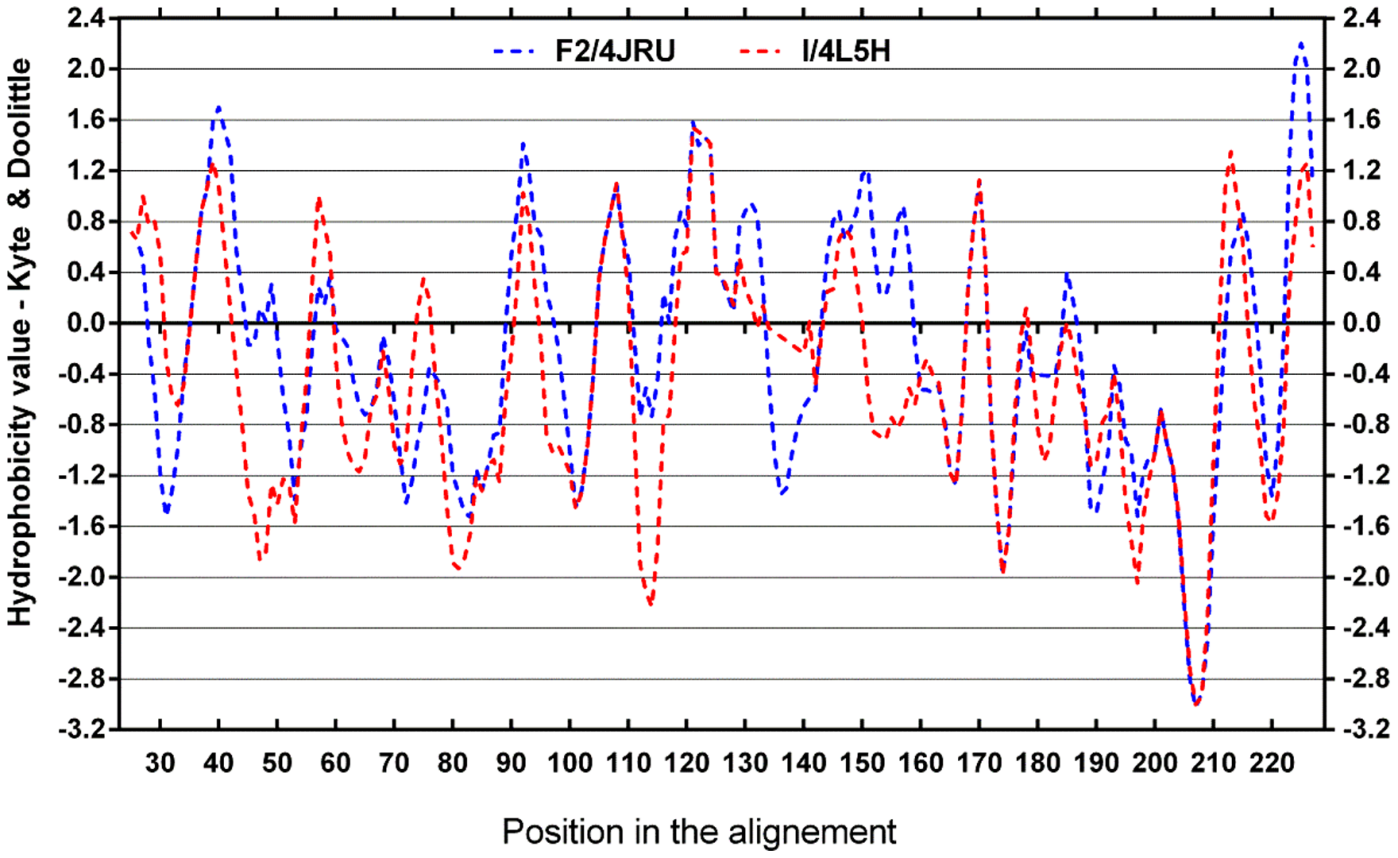
Comparison of the hydrophobicity of proteins F2/4JRU and I/4L5H. Hydrophobicity plot based on Kyte & Doolittle's scale for the alignment of proteins F2/4JRU (blue) and I/4L5H (red) obtained with AlignMe software http://www.bioinfo.mpg.de/AlignMe/AlignMe.html [56]. Window size used for analysis: 9 residues.

Because differences in protein hydrophobicity were seen during the HIC purification step (Table 3), the hydrophobicities of proteins F2/4JRU and I/4L5H were compared. Differences in hydrophobicity in certain regions can lead to different hydrophobic binding sites being exposed upon heat unfolding, with consequences in the aggregation behaviour of the proteins (Figure 6).

Figure 6 shows that there are several regions of the proteins having substantial differences in hydrophobicity. In particular there are 4 main regions where I/4L5H is more hydrophilic than F2/4JRU: in β-strands 3 and 4 (residues 45–55) I/4L5H reaches values double those of F2/4JRU (−1.6 vs −0.8); in β-strands 8 and 9 (residues 110–120) I/4L5H is very hydrophilic (−1.6) while F2/4JRU only reaches −0.4; in β-strand 11 and α–helix 1 (residues 145–160) I/4L5H is also somewhat hydrophilic (hydrophobicity of −1.2), while F2/4JRU is hydrophobic (hydrophobicity up to 1.5); in β-strand 15 (residues 220–225) I/4L5H is again hydrophilic while F2/4JRU is hydrophobic. The grand average of hydrophobicity of the two proteins is −0.206 for F2/4JRU and −0.438 for I/4L5H [43], which agrees with findings from the HIC purification (Table 3). Based on these data it can be hypothesised that differences in solubility shown by these two classes of proteins could be directly linked to the differences in hydrophobicity observed in different regions of the proteins, with the more hydrophilic protein (I/4L5H) being also the more soluble and therefore heat-stable, confirming data shown in Table 3 and Figure 4.

## Discussion

### The role of TLPs in wine haze formation

The proteins responsible for haze formation in wines are derived from grape juice [44]. Several authors have reported changes occurring to the protein content and relative proportion of different protein classes during fermentation [45]–[47], with the less soluble proteins tending to precipitate. Among the proteins that ‘survive’ the fermentation process, thaumatin-like proteins are generally the most represented class found in wine along with chitinases [48]. The role of grape thaumatin-like proteins in the formation of protein haze during wine storage is somewhat controversial. Initial studies on the identification of wine proteins responsible for haze formation indicated that TLPs, as well as chitinases, were the main proteins responsible [5], [48]. This assumption led to these two classes of proteins being generally discussed in parallel and associated in term of their importance in haze formation in wines [44]. The more recent development of a method for their purification in large quantities and high purity [9], facilitated further characterisation of these proteins and elucidation of the roles played by different PR-proteins in the mechanism of haze formation. It is now established that there are differences in the mechanism of haze formation between chitinases and TLPs. Chitinases have been shown to have lower heat stability than TLPs, as well as an irreversible unfolding behaviour, while TLPs have a partially reversible unfolding behaviour and generally return to a 3D state after cooling [10], [11]. These lines of evidence support the hypothesis that chitinases are more prone to form visible haze in wine than thaumatin-like proteins. However, this conclusion does not fully match with the evidence available in more recent literature in which TLPs have been reported to be present in wine hazes [2], [15], [16], [46]. A possible solution for the discrepancies noted in the literature was recently found, as it was firstly reported that different grape TLPs can have a different hazing behaviour and therefore heat stability and aggregation characteristics [12]. Indeed, some TLPs have been shown to have a behaviour more similar to that of chitinases since they have a lower unfolding temperature than other TLPs, less reversibility of their unfolding and a tendency to aggregate and form haze upon unfolding, while other TLPs do not [10]–[13]. This theory is supported by the result shown in Figure 4, in which, despite the sequence and structural similarities, only F2/4JRU formed haze upon heat test.

### The structural differences among TLPs leading to their different heat stability

Isoform differences in heat stability have practical and economic ramifications for winemaking. However, from the structures of the three proteins, it is clear that they only significantly differ in two loop regions (Figure 3). In particular, the loop in Figure 3E is located between β-strands 9 and 10. In this region F2/4JRU is more hydrophilic than I/4L5H (Figure 6, residues 135–141). However, flanking regions show major differences, with β-strands 7, 8 and 14 of protein F2/4JRU being significantly more hydrophobic than the same regions of I/4L5H, while β-strand 9 was more hydrophobic for F2/4JRU and β-strand 10 was similar between proteins. The second differing loop (Figure 3F) located between helixes α3 and α4 did not show differences in hydrophobicity (Figure 6, residues 176–177), and neither did neighboring regions. From these data it seems that the main solubility and aggregation differences are due to the variation in the loop between β-strands 9 and 10 (Figure 3E). It is likely that this loop, despite having similar charge and hydrophobicity in F2/4JRU and I/4L5H, following the unfolding event can result in protein destabilisation. The mechanism of this destabilisation is probably related to higher levels of exposure that this loop has in protein F2/4JRU, when compare to I/4L5H. Since this loop is located in the extensive β-sheet region in the core domain of the protein, and since it is more hydrophilic than the same loop in protein I/4L5H, upon unfolding it could expose the more hydrophobic neighbouring regions (β-strands 7, 8 and 14 of protein F2/4JRU), thus favouring protein aggregation. In this way, the refolding of F2/4JRU would be hindered, aggregation would take place, and haze in wine would be formed. The opposite is true for this loop in protein I/4L5H which is much shorter and less exposed, but also hydrophobic, while the neighbouring β-strands are more hydrophilic relative to F2/4JRU. Therefore, even upon unfolding aggregation is much less likely to occur allowing the protein to return to a native state without contributing to haze formation in wines.

The forces involved in protein aggregation in thaumatin-like proteins have not been experimentally investigated. However, it is likely that the interactions are similar to those observed with proteins that have been demonstrated to form amyloid aggregates. In these cases, the aggregation involves hydrogen bonding with the formation of β-sheet between the aggregating polypeptides [49]. In these examples the destabilization of the native protein structure by either amino acid substitution or reduction of disulfide bonds increases the propensity for proteins to aggregate [49]. Therefore, the presence of the disulfide bridge in this loop is probably the key for the unfolding/refolding behaviour of this area (residues 140–213, connecting the loop to β-strand 13). White wines are typically produced in reducing conditions and with SO_2_ added to prevent faults due to oxidation. These conditions would exacerbate haze formation, particularly for protein F2/4JRU as the S-S bond shown in Figure 3E could be cleaved, thus allowing hydrophobic aggregation to take place. This hypothesis is in agreement with Marangon et al. [7] who found that the presence of a reducing agent (DTT) during the heat-induced unfolding of wine proteins in a model system caused the irreversible destabilisation of the protein due to the exposure of their buried hydrophobic binding sites. The authors also suggested that this mechanism seemed to be typical of the TLPs, a fact that is now supported by the structural data discussed above.

### Structural information can lead to a change in winemaking practices

For the first time the structure of proteins responsible for haze formation in wines have been elucidated. Three TLP isoforms, belonging to two slightly different fold categories had different unfolding temperature and aggregation characteristics, with F2/4JRU being more unstable than I/4L5H and H2/4MBT. From the detailed analysis of their structural features it appears that the difference in function among proteins is attributable to the differences observed in a loop located between β-strands 9 and 10 and of the neighbouring regions (Figure 3E). The differences potentially produce higher or lower degrees of protein destabilisation once unfolding takes place, thus affecting protein aggregation and therefore haze formation in wines. As the heat instability of F2/4JRU is most likely due to this exposed loop containing a disulphide bridge with flanking hydrophobic regions, the reduction of this S-S bridge could lead to the destabilisation of the protein and subsequent aggregation, hence resulting in haze formation in wines. From a practical perspective, in winemaking this new piece of fundamental information could be exploited by inducing this protein destabilisation/aggregation process earlier in the winemaking process, so that settling and filtration after protein destabilization would result in the removal of the heat unstable protein aggregates before bottling. Aggregation of heat unstable TLPs could be induced by heat application, but winemakers are generally reluctant to pasteurize juices or wines because of possible negative impact on the wine sensory characteristics. Other options would therefore need to be developed.

In addition, if the role of SO_2_ as a reducing agent favouring protein unfolding in wines is confirmed, future research should look into the possible development of non-reducing preservatives for the wine industry as they could reduce the incidence of haze formation from these proteins and lead to a reduced need of protein fining in wines. Alternatively, rather than trying to avoid TLP aggregation, another approach would be trying to encourage it with the use of alternative reducing agents, although this would not be very practical for the beverage industry as reducing agents are generally not food grade.

The elucidation of the structural characteristics of the grape TLPs could also be used to find another practical solution to the problem of wine hazing: proteolysis. Although proteolytic enzymes able to degrade TLPs once TLPs are unfolded at elevated temperatures have been discovered [50], an enzyme capable of doing this at the lower temperatures usual for winemaking is more elusive. Van Sluyter et al. [51] found that a protease from a grape pathogen partially removes TLPs under normal winemaking conditions. However, the mechanism of removal has not been characterized. The information described in this paper will make the quest for finding such an enzyme more targeted, as researchers could use a modeling approach to screen for enzymes able to cleave the grape TLPs. The candidates showing a theoretical ability to cleave the TLPs could then be selected on the basis of their characteristics (e.g., if they are allowed for winemaking and/or if they are food grade) so that scale up experiments could be carried out on the selected enzymes. This approach, in our opinion, is more likely to succeed than the non-targeted approaches performed so far [50], [52]–[54].

If an enzyme were to be found following this approach, even if it were not able to fully degrade TLPs, it could still represent a solution if it were able to cleave the loop responsible for heat instability, as this might trigger the onset of protein aggregation by aiding the destabilisation of the protein's 3D structure. Therefore, if this enzyme was added early in the winemaking process, TLP aggregation might be favored, allowing protein aggregates to be eliminated earlier via racking or filtration operations.

The availability of structural information on haze forming proteins could result in the identification of viable enzymes for the prevention of wine hazing, resulting in an important change in the winemaking stabilization practices. The advantages of moving away from bentonite as the method for preventing wine hazing could have large impacts both economically and environmentally.

## Supporting Information

Table S1 **Purified protein sequences.** Protein designations according to Van Sluyter et al. [9].
(DOCX)

Table S2 **Crystallization conditions.**
(DOCX)

Table S3 **Protein sequence similarity analysis.** Pairwise alignment of the 4 proteins performed with EMBOSS Needle Pairwise Sequence Alignment (http://www.ebi.ac.uk/Tools/psa/emboss_needle/) [57].
(DOCX)
