# Supplementary material for: Structure of Haze Forming Proteins in White Wines: Vitis vinifera Thaumatin-Like Proteins
Source: PLoS One. 2014 Dec 2;9(12):e113757. doi: 10.1371/journal.pone.0113757 (PMC4252030; doi:10.1371/journal.pone.0113757)
Supplement: Table S1 — Purified protein sequences. Protein designations according to Van Sluyter et al. [9]. (DOCX) [file pone.0113757.s001.docx]

| **Protein designation**  **(PDB ID)** | **Complete amino acid sequence** |
| --- | --- |
| **F2**  **(4JRU)** | ATFNIQNHCSYTVWAAAVPGGGMQLGSGQSWSLNVNAGTTGGRVWARTNCNFDASGNGKCETGDCGGLLQCTAYGTPPNTLAEFALNQFSNLDFFDISLVDGFNVPMAFNPTSNGCTRGISCTADIVGECPAALKTTGGCNNPCTVFKTDEYCCNSGSCSTDYSRFFKTRCPDAYSYPKDDQTSTFTCTAGTNYEVVFCP |
| **I**  **(4L5H)** | ATFDILNKCTYTVWAAASPGGGRRLDSGQSWTITVNPGTTNARIWGRTSCTFDANGRGKCETGDCNGLLECQGYGSPPNTLAEFALNQPNNLDYIDISLVDGFNIPMDFSGCRGIQCSVDINGQCPSELKAPGGCNNPCTVFKTNEYCCTDGPGSCGPTTYSKFFKDRCPDAYSYPQDDKTSLFTCPSGTNYKVTFCP |
| **H2**  **(4MBT)** | ATFDILNKCTYTVWAAASPGGGRRLDSGQSWTITVNPGTTNARIWGRTSCTFDANGRGKCETGDCNGLLECQGYGSPPNTLAEFALNQPNNLDYIDISLVDGFNIPMDFSGCRGIQCSVDINGQCPSELKAPGGCNNPCTVFKTNEYCCTDGPGSCGPTTYSKFFKDRCPDAYSYPQDDKTSLFTCPSGTNYKVTFCP |
