# Supplementary material for: Structure of Haze Forming Proteins in White Wines: Vitis vinifera Thaumatin-Like Proteins
Source: PLoS One. 2014 Dec 2;9(12):e113757. doi: 10.1371/journal.pone.0113757 (PMC4252030; doi:10.1371/journal.pone.0113757)
Supplement: Table S2 — Crystallization conditions. (DOCX) [file pone.0113757.s002.docx]

|  | **F2/4JRU** | **I/4L5H** | **H2/4MBT** |
| --- | --- | --- | --- |
| **Method** | hanging-drop vapor diffusion | hanging-drop vapor diffusion | hanging-drop vapor diffusion |
| **Plate type** | 24 wells Intelliplate | 24 wells Intelliplate | 24 wells Intelliplate |
| **Temperature (K)** | 293 | 293 | 293 |
| **Protein Concentration (mg/mL)** | 12 | 28 | 20 |
| **Buffer composition of protein solution** | 5 mM formic acid, 10 mM NaCl | 5 mM formic acid, 10 mM NaCl | 5 mM formic acid, 10 mM NaCl |
| **Composition of reservoir solution** | 0.1M Na acetate pH 4.6, 6% PEG 4000, 10 mM MgCl_2_ | 2M Mg acetate, 0.1 M Na acetate, pH 4.6 | 2.58 M Mg acetate, 0.2 M MES |
| **Volume and ratio of drop** | 1 µL (1:1) | 1 µL (1:1) | 1 µL (1:1) |
| **Volume of reservoir** | 500 µL | 500 µL | 500 µL |
