# Supplementary material for: Structure of Haze Forming Proteins in White Wines: Vitis vinifera Thaumatin-Like Proteins
Source: PLoS One. 2014 Dec 2;9(12):e113757. doi: 10.1371/journal.pone.0113757 (PMC4252030; doi:10.1371/journal.pone.0113757)
Supplement: Table S3 — Protein sequence similarity analysis. Pairwise alignment of the 4 proteins performed with EMBOSS Needle Pairwise Sequence Alignment (http://www.ebi.ac.uk/Tools/psa/emboss_needle/) [57]. (DOCX) [file pone.0113757.s003.docx]

| 1^st^ protein | 2^nd^ protein | Length | Identity | Similarity | Gaps | Score |
| --- | --- | --- | --- | --- | --- | --- |
| *1Z3Q* | *F2/4JRU* | 201 | 160/201  (79.6%) | 174/201  (86.6%) | 1/201  (0.5%) | 931.0 |
| *1Z3Q* | *I/4L5H* | 202 | 146/202  (72.3%) | 170/202  (84.2%) | 6/202  (3.0%) | 868.0 |
| *1Z3Q* | *H2/4MBT* | 202 | 146/202  (72.3%) | 170/202  (84.2%) | 6/202  (3.0%) | 868.0 |
| *F2/4JRU* | *I/4L5H* | 203 | 140/203  (69.0%) | 163/203  (80.3%) | 7/203  (3.4%) | 804.0 |
| *F2/4JRU* | *H2/4MBT* | 203 | 140/203  (69.0%) | 163/203  (80.3%) | 7/203  (3.4%) | 804.0 |
| *I/4L5H* | *H2/4MBT* | 198 | 198/198  (100.0%) | 198/198  (100.0%) | 0/198  (0.0%) | 1131.0 |
